# mTOR in cerebrovascular disease

**DOI:** 10.18632/aging.101869

**Published:** 2019-03-13

**Authors:** Candice E. Van Skike, Veronica Galvan

**Affiliations:** 1 Department of Cellular and Integrative Physiology, University of Texas Health San Antonio, TX 78229, USA; 2 Barshop Institute for Longevity and Aging Studies and Glenn Biggs Institute for Alzheimer’s and Neurodegenerative Diseases, University of Texas Health San Antonio, TX 78229, USA; 3 Department of Veterans Affairs, South Texas Veterans Health Care System, San Antonio, TX 78229, USA

**Keywords:** mTOR, Alzheimer’s disease, vascular cognitive impairment, atherosclerosis, blood-brain barrier

Recent research suggests cerebrovascular dysfunction is an early pathological event in Alzheimer’s disease (AD) progression [1]. Our lab and others have demonstrated that the mechanistic/mammalian target of rapamycin (mTOR) mediates several different aspects of cerebrovascular dysfunction in AD models, including blood-brain barrier (BBB) breakdown, cerebral hypoperfusion, reduced cerebrovascular reactivity, and impaired neurovascular coupling (please see [2] for a review of these mechanisms). However, recent data suggests mTOR activity may contribute to cerebrovascular dysfunction arising from primary vascular impairment [3], and therefore the mTOR pathway may be a critical effector of cerebrovascular dysfunction in disorders of both neurological and non-neurological etiology.

We have recently reported cognitive impairments in high-fat diet fed LDL receptor null (HFD-LDLR^-/-^) mice, a model of atherosclerosis [3]. We propose that this model recapitulates critical aspects of vascular cognitive impairment (VCI) because cognitive impairment in this model has a purely vascular etiology. In addition, we have shown that VCI in HFD-LDLR^-/-^ mice is dependent on mTOR-driven cerebrovascular dysfunction, including cerebral hypoperfusion evidenced by reduced cerebral blood flow [3], and BBB breakdown [4]. Attenuation of mTOR via rapamycin restores cerebrovascular dysfunction in HFD-LDLR^-/-^ mice [3,4], and is associated with improved cognitive outcomes [3].

Additionally, mTOR inhibition has been shown to reduce atherosclerotic lesions in the aortic arch of HFD-LDLR^-/-^ mice, even though hypercholesterolemia was not reduced [3], indicating that some of the improvements in cerebrovascular function may have originated from effects mediated through enhanced cardiovascular factors. Therefore, it is likely that restoration of cerebral blood flow by mTOR attenuation is due to the combined amelioration of both cerebrovascular (loss of brain vascular density, BBB breakdown) and cardiovascular pathologies in the HFD-LDLR^-/-^ model of VCI.

These data therefore suggest that the mTOR pathway drives both cardiovascular and cerebrovascular dysfunction in a model of cognitive impairment where the primary injury is cardiovascular pathophysiology. While these studies do not rule out that cerebrovascular impairments may be secondary to cardiovascular dysfunction, a recent study indicates mTOR attenuation via caloric restriction improves cerebral blood flow in 6 month old C57BL/6N mice, presumably devoid of cardiovascular damage [5, 6]. This suggests that cerebral blood flow can be enhanced by mTOR attenuation even in the absence of vascular pathology. Therefore, when taken together, our recent data [3,4] indicate a primary role of mTOR in cerebrovascular dysfunction of different etiologies, independent of cardiovascular compromise. That HFD-LDLR^-/-^ mice display profound cerebral hypoperfusion, however, implies a mechanistic link between cardiovascular and cerebrovascular function and dysfunction in this model. This is consistent with the observation that the presence of cardiovascular risk factors in humans is associated with negative neurological outcomes, such as brain hypoperfusion and cognitive impairments including VCI and AD [7]. Taken together, these observations lend support to the concept that HFD-LDLR^-/-^ mice may constitute a useful model of VCI.

To summarize, research indicates that cerebrovascular dysfunction in AD is dependent on mTOR activity (reviewed in [2]). However, we have recently shown that mTOR-dependent cerebrovascular dysfunction includes an mTOR-dependent component where the kinase drives cardiovascular pathology, impacting cerebrovascular function [3]. It is likely that the cerebrovascular deficits associated with atherosclerosis are secondary to cardiovascular disease based on the disease presentation and progression in humans, however to our knowledge this has not yet been defined in the HFD-LDLR^-/-^ mouse model of atherosclerosis. Future studies are warranted to delineate the relationship between mTOR-dependent cardiovascular and cerebrovascular dysfunction and the potential for interventions that may act on both local and systemic mechanisms to alleviate disease processes. Therefore, mTOR and effectors of the mTOR pathway may constitute powerful novel pharmacotheraputic targets in disorders that have cardiovascular and cerebrovascular dysfunction as a common etiology.
